# Decreased cytokine production by mononuclear cells after severe gram-negative infections: early clinical signs and association with final outcome

**DOI:** 10.1186/s13054-017-1625-1

**Published:** 2017-03-09

**Authors:** Nikolaos Antonakos, Thomas Tsaganos, Volker Oberle, Iraklis Tsangaris, Malvina Lada, Aikaterini Pistiki, Nikolaos Machairas, Maria Souli, Michael Bauer, Evangelos J. Giamarellos-Bourboulis

**Affiliations:** 1 4th Department of Internal Medicine, Medical School, National and Kapodistrian University of Athens, Athens, Greece; 2 Department of Clinical Chemistry and Laboratory Medicine, Jena University Hospital, Jena, Germany; 3 Centre for Sepsis Control and Care, Jena University Hospital, Jena, Germany; 4 2nd Department of Critical Care Medicine, Medical School, National and Kapodistrian University of Athens, Athens, Greece; 5 2nd Department of Internal Medicine, Sismanogleion Athens General Hospital, Athens, Greece; 6 Department of Anaesthesiology and Intensive Care, Jena University Hospital, Jena, Germany

**Keywords:** Sepsis, Immunosuppression, Survival, Prediction

## Abstract

**Background:**

Failure of circulating monocytes for adequate cytokine production is a trait of sepsis-induced immunosuppression; however, its duration and association with final outcome are poorly understood.

**Methods:**

We conducted a substudy of a large randomised clinical trial. Peripheral blood mononuclear cells (PBMCs) were isolated within the first 24 h from the onset of systemic inflammatory response syndrome in 95 patients with microbiologically confirmed or clinically suspected gram-negative infections. Isolation was repeated on days 3, 7 and 10. PBMCs were stimulated for cytokine production. The study endpoints were the differences between survivors and non-survivors, the persistence of immunosuppression, and determination of admission clinical signs that can lead to early identification of the likelihood of immunosuppression.

**Results:**

PBMCs of survivors produced significantly greater concentrations of tumour necrosis factor-α (TNF-α), interleukin (IL)-6, IL-8, IL-10, interferon-γ and granulocyte-macrophage colony-stimulating factor after day 3. Using ROC analysis, we found that TNF-α production less than 250 pg/ml after lipopolysaccharide stimulation on day 3 could discriminate patients from healthy control subjects; this was associated with a 5.18 OR of having an unfavourable outcome (*p* = 0.046). This trait persisted as long as day 10. Logistic regression analysis showed that cardiovascular failure on admission was the only independent predictor of defective TNF-α production on day 3.

**Conclusions:**

Defective TNF-α production is a major trait of sepsis-induced immunosuppression. It is associated with significant risk for unfavourable outcome and persists until day 10. Cardiovascular failure on admission is predictive of defective TNF-α production during follow-up.

**Trial registration:**

ClinicalTrials.gov identifier: NCT01223690. Registered on 18 October 2010.

**Electronic supplementary material:**

The online version of this article (doi:10.1186/s13054-017-1625-1) contains supplementary material, which is available to authorized users.

## Background

Sepsis is the leading cause of death in critically ill patients. Mortality can reach up to 70% of patients with septic shock and up to 80% of patients with multiple organ dysfunction [1]. This great mortality reflects our poor understanding of sepsis for recognition and management as well as of the mechanisms of its pathogenesis and pathophysiology. Although traditionally conceived of as an exaggerated reaction of the host to a microbial insult, one current suggestion of the immunobiology of sepsis is that, inside the septic host, pro-inflammatory and anti-inflammatory phenomena co-exist. The phenomena may involve both hyper-production of pro-inflammatory cytokines by the innate immune system and impaired innate and adaptive immune responses [2]. Impaired immune responses are characterised by enhanced apoptosis and dysfunction of lymphocytes, impaired phagocyte functions and decreased ex vivo cytokine production [2–4].

Early patient death may result from exaggerated pro-inflammatory responses [2]. However, late death may be related to a late anti-inflammatory phase sometimes called *sepsis-induced immunosuppression*. In their study, Boomer et al. explored the function of splenocytes isolated from early cadavers of severe sepsis and compared them with those from brain-dead subjects with multiple injuries. A clear-cut down-regulation of both innate and adaptive immune responses was found, leading to the assumption that sepsis-induced immunosuppression is the driver towards unfavourable outcome [5].

The findings of the study by Boomer et al. [5] generated some important questions:

- How is cytokine production associated with final outcome?
- How long does down-regulation of sepsis last?
- Which clinical signs on admission can help discriminate the patient who is entering into the immunosuppression stage?

A proper answer to these questions can lead to appropriate application of drugs with the aim of reversing immunosuppression of sepsis. In the present study, we used serial stimulation of circulating mononuclear cells during patient follow-up to seek answers to these questions.

## Methods

### Study design

A prospective, randomised clinical trial (ClinicalTrials.gov identifier NCT01223690) was conducted during the period from July 2007 to April 2011 in six departments in Greece to assess the efficacy and safety of intravenous clarithromycin over placebo for patients with microbiologically documented gram-negative infections or with infections at high clinical risk of being of gram-negative origin [6]. During this trial, peripheral blood mononuclear cells (PBMCs) were isolated at serial time intervals for cytokine stimulation from a subgroup of patients, and because all-cause mortality was similar in placebo- and clarithromycin-treated patients, results were analysed together. The subgroup study design was approved by the ethics committee of Attikon University Hospital (license 6/18-05-2011), and written informed consent was obtained from participants’ first-degree relatives.

Inclusion criteria for this subgroup of patients were (a) age 18 years or older; (b) written informed consent provided by first-degree relatives; (c) presence of at least two signs of the systemic inflammatory response syndrome (SIRS); (d) presence of acute pyelonephritis, acute intra-abdominal infection or primary bacteraemia caused by gram-negative bacteria; and (e) start of blood sampling within the first 24 h from the onset of signs of SIRS. Exclusion criteria were (a) neutropenia due to causes other than SIRS, defined as an absolute number of neutrophils less than 1000/mm^3^; (b) diagnosis of HIV infection; and (c) corticosteroid treatment over the last 30 days in doses greater than 0.4 mg/kg prednisone or equivalent.

Definitions of acute pyelonephritis, acute intra-abdominal infections, primary gram-negative bacteraemia and organ failure have been published [6]. The following data were recorded for every patient for 28 days: age, sex, previous medical history, vital signs, clinical signs, type of infection, type of failing organ, microbiology, laboratory findings, Acute Physiology and Chronic Health Evaluation (APACHE) II score, Sequential Organ Failure Assessment (SOFA) score and final outcome. A retrospective evaluation of these patients showed that they all met the new Sepsis-3 definitions of admission SOFA score greater than or equal to 2 for patients admitted at the emergency department or an increase of admission SOFA score greater than or equal to 2 for patients developing sepsis after hospital admission [7]. Cardiovascular (CV) failure in patients was defined as any persisting systolic blood pressure below 90 mmHg despite restoration of negative fluid balance that necessitated the administration of vasopressors. A retrospective evaluation of patients with CV failure showed that they all met the new Sepsis-3 definition of septic shock [7].

### Laboratory investigation

All laboratory investigation was run in the central laboratory of the 4th Department of Internal Medicine at Attikon University Hospital, Athens, Greece. The time interval from blood sampling until processing was a maximum of 1 h. Twenty millilitres of heparinised blood was collected after venepuncture of one forearm vein under aseptic conditions during the first 24 h from the onset of signs of SIRS (day 1) and repeated on days 3, 7 and 10. PBMCs were separated after gradient centrifugation of the blood over Ficoll-Hypaque density gradient (Biochrom, Berlin, Germany). After three washings in ice-cold PBS, pH 7.2 (Biochrom), PBMCs were counted using a Neubauer plate with trypan blue exclusion of dead cells. PBMCs were then stimulated in duplicate at a density of 5 × 10^6^ cells/ml at 37 °C in a 5% CO_2_ atmosphere in RPMI 1640 medium enriched with 2 mM of l-glutamine, 100 U/ml penicillin G, 100 mg/ml gentamicin and 10 mM pyruvate with or without 10 ng/ml lipopolysaccharide (LPS) derived from *Escherichia coli* O55:B5 (Sigma-Aldrich, St. Louis, MO, USA), and 5 μg/ml Pam_3_Cys-SKKK (EMC Microcollections, Tübingen, Germany). After incubation for 24 or 48 h, plates were centrifuged at 800 × *g* for 7 minutes, and the supernatants were collected and stored at −70 °C until assayed. Cytokines were measured with a Bio-Plex Pro Human Cytokine Panel on a Bio-Rad Luminex 200 suspension array system (Bio-Rad Laboratories, Hercules, CA, USA). The measurements were carried out according to the manufacturer’s instructions. All samples were measured in duplicate. Every plate had its own standard curve built from data also measured in duplicate. The cytokines used were tumour necrosis factor-α (TNF-α), interleukin (IL)-6 and IL-8 with supernatants coming from the 24-h incubation of the plates, as well as IL-4, IL-10, IL-12, interferon-γ (IFN-γ) and granulocyte-macrophage colony-stimulating factor (GM-CSF) using supernatants coming from the 48-h incubation of the plates. The lower limit of detection was 0.5 pg/ml (Additional file 1: Table S1). PBMCs were also isolated from 16 healthy individuals during the 2007–2011 period and stimulated as described above in the absence and presence of LPS. TNF-α was measured as described above to be used as control measurements.

### Statistical analysis

Results were expressed as mean ± SE. Patients were divided into survivors and non-survivors on the basis of their 28-day outcome. Comparison of each cytokine between healthy control subjects and patients as well as between survivors and non-survivors was done using the Mann-Whitney *U* test. ROC curve analysis was done to identify TNF-α production on day 3 that could significantly differentiate between patients and healthy control subjects. Using this ROC curve, a cut-off cytokine concentration with more than 80% sensitivity for this discrimination was defined. Patients above this cut-off were considered to have adequate TNF-α production by circulating PBMCs on day 3; those below this cut-off were considered to have defective TNF-α production by circulating PBMCs on day 3. ORs and 95% CIs for death between patients with adequate and defective TNF-α production on day 3 were determined using Mantel-Haenszel statistics. In order to identify which patients’ characteristics at admission might be predictive of defective TNF-α production on day 3, admission characteristics of patients with adequate and defective TNF-α production on day 3 were compared. Comparisons were done with Student’s *t* test for quantitative variables and the chi-square test for qualitative variables. A step-wise forward logistic regression analysis was conducted with defective TNF-α production on day 3 as the dependent variable; admission characteristics with differences at a *p* value below 0.10 were entered into the equation as independent variables; and ORs and 95% CIs were determined. Any *p* value after adjustment for multiple comparisons according to the method of Bonferroni was considered significant.

## Results

**Fig. 1:**
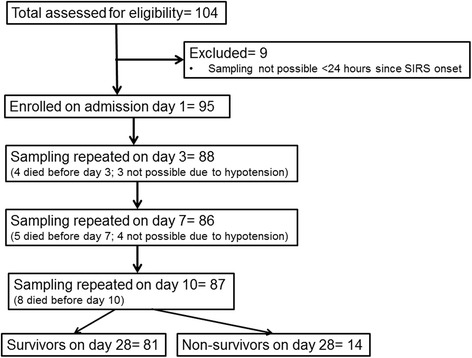
Study flowchart. *SIRS* Systemic inflammatory response syndrome

From among the total enrolled patients in the clinical trial, 95 patients participated in the substudy. The study flowchart is presented in Fig. 1, and the demographic and clinical characteristics of enrolled patients are displayed in Table 1.

**Table 1:** Demographic and clinical characteristics of the 95 patients enrolled in the study

Characteristics	Data
Males/females, *n* (%)	52 (54.7%)/43 (45.3%)
Age, years, mean ± SD	68.8 ± 17.0
APACHE II score, mean ± SD	13.83 ± 7.86
PaO_2_/FiO_2_, mmHg, mean ± SD	307.6 ± 129.4
White blood cells, count/mm^3^, mean ± SD	14,735.0 ± 7717.0
C-reactive protein, mg/L mean ± SD	137.2 ± 97.3
Type of infection	
Acute pyelonephritis, *n* (%)	45 (47.4%)
Acute intra-abdominal infection, *n* (%)	31 (32.6%)
Primary gram-negative bacteraemia, *n* (%)	19 (20.0%)
Failing organs on enrolment, *n* (%)	25 (26.3%)
Acute respiratory distress syndrome, *n* (%)	16 (16.8%)
Acute coagulopathy	13 (13.7%)
Acute kidney injury	12 (12.6%)
Cardiovascular failure	12 (12.6%)
Isolated pathogens in blood and/or urine, *n* (%)	
*Escherichia coli*	25 (26.3%)
*Klebsiella pneumoniae*	8 (8.4%)
*Proteus mirabilis*	5 (5.3%)
*Pseudomonas aeruginosa*	3 (3.2%)
*Acinetobacter baumannii*	3 (3.2%)
Other gram-negative bacteria	8 (8.4%)
Co-existing disorders, *n* (%)	
Type 2 diabetes mellitus	29 (30.5%)
Solid tumour malignancy	22 (23.2%)
Chronic renal disease	17 (17.9%)
Chronic obstructive pulmonary disease	15 (15.8%)
Chronic heart failure	9 (9.5%)
Predisposing factors, *n* (%)	
Cerebral stroke	14 (14.7%)
Nephrolithiasis	11 (11.6%)
Gallstones	12 (12.6%)
Multiple injuries	3 (3.2%)

*APACHE* Acute Physiology and Chronic Health Evaluation, *PaO*_*2*_ */FiO*_*2*_ Ratio of partial pressure arterial oxygen and fraction of inspired oxygen

**Fig. 2:**
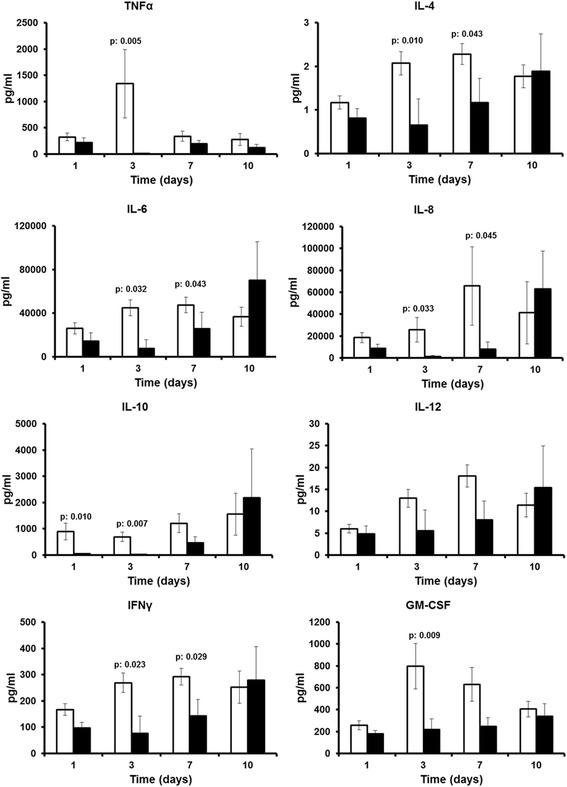
Cytokine production after stimulation with bacterial endotoxin. Cytokine production from medium-stimulated cells was below the limit of detection. *White bars* represent survivors and *black bars* non-survivors after 28 days of follow-up. *p* Values indicate statistically significant differences between survivors and non-survivors at the indicated time intervals. Non-significant differences are now shown. *GM-CSF* Granulocyte-macrophage colony-stimulating factor, *IFN-*γ Interferon-γ, *IL* Interleukin, *TNF-*α Tumour necrosis factor-α

**Fig. 3:**
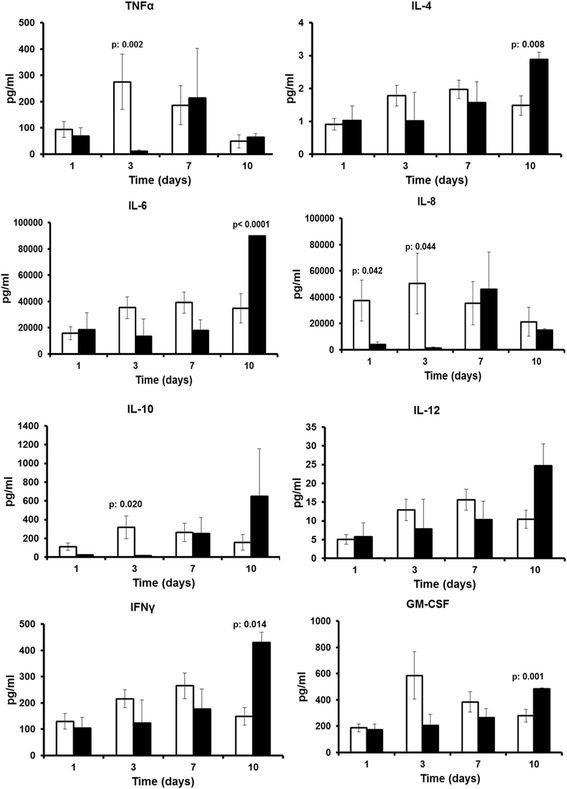
Cytokine production after stimulation with Pam_3_Cys. Cytokine production from medium-stimulated cells was below the limit of detection. *White bars* represent survivors and *black bars* non-survivors after 28 days of follow-up. *p* Values indicate statistically significant differences between survivors and non-survivors at the indicated time intervals. Non-significant differences are now shown. *GM-CSF* Granulocyte-macrophage colony-stimulating factor, *IFN-*γ Interferon-γ, *IL* Interleukin, *TNF-*α Tumour necrosis factor-α

Patients were divided into survivors and non-survivors on the basis of their 28-day outcome. Figure 2 shows the production of cytokines from PBMCs of survivors and non-survivors on admission day 1 and on serial days 3, 7 and 10 after stimulation with LPS. Cytokines in supernatants of medium-treated cells were below the limit of detection. Although cytokine production by PBMCs after LPS stimulation was similar for survivors and non-survivors on day 1 with the exception of IL-10, PBMCs of survivors produced significantly greater concentrations of TNF-α, IL-4, IL-6, IL-8, IL-10, IFN-γ and GM-CSF than PBMCs of non-survivors starting on day 3 and mainly shown on day 7. The respective increase after Pam_3_Cys stimulation was shown for a lower number of cytokines, mainly TNF-α and IL-10 (Fig. 3).

**Fig. 4:**
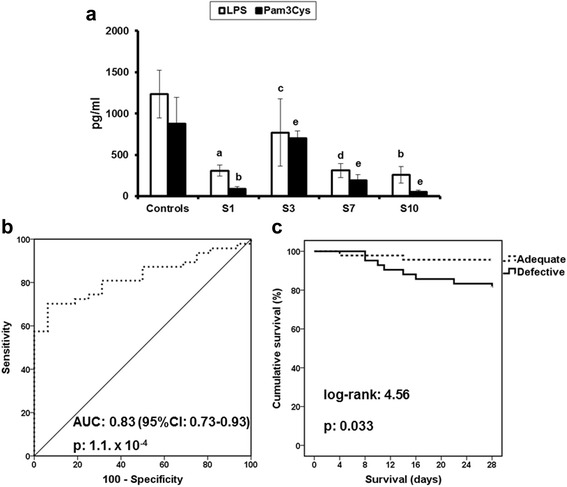
TNF-α production by LPS-stimulated PBMCs as an index of sepsis-induced immunosuppression. **a** Comparative production of TNF-α by PBMCs from healthy control subjects and patients after stimulation with LPS and Pam_3_Cys. The exact *p* values after correction for multiple testing are provided: ^a^9.8 × 10^−4^; ^b^2.5 × 10^−3^; ^c^7.6 × 10^−4^; ^d^5.7 × 10^−4^; ^e^non-significant. **b** ROC curve analysis to identify a cut-off concentration of TNF-α produced by PBMCs after stimulation with LPS on day 3 between patients and healthy control subjects. **c** Survival analysis between patients with production of TNF-α less than 250 pg/ml (defective, *n* = 42) and patients with production of TNF-α more than 250 pg/ml (adequate, *n* = 46). *p* Values of significance are provided. *LPS* Lipopolysaccharide, *PBMC* Peripheral blood mononuclear cell, *TNF-*α Tumour necrosis factor-α

These findings led us to hypothesise that survival is associated with improved cytokine production for TNF-α because TNF-α was the only cytokine that was produced early from PBMCs of survivors after stimulation with both stimuli used. TNF-α production by PBMCs of patients was significantly lower than that by PBMCs of healthy control subjects during all days of follow-up when PBMCs were stimulated with LPS; this was not the case when PBMCs were stimulated with Pam_3_Cys, where significant differences ceased to exist after day 3 (Fig. 4a). This made us consider that LPS is a more sensitive stimulus to indicate sepsis-induced immunosuppression, which lasted until day 10. When separate comparisons were done between TNF-α produced by PBMCs of healthy control subjects and by survivors and non-survivors of sepsis, production of TNF-α was significantly lower in both survivors and non-survivors on days 3, 7 and 10 when LPS was used as a stimulus; this was not the case when Pam_3_Cys was used as a stimulus (Additional file 2: Table S2).

Next, we designed a ROC curve to discriminate TNF-α production by PBMCs after LPS stimulation on day 3 between patients and healthy volunteers. Generated ROC curves for TNF-α production after LPS stimulation provided a statistically significant AUC (Fig. 4b). Co-ordinate points of the ROC curve showed that TNF-α production after stimulation with LPS less than 250 pg/ml could discriminate patients from healthy volunteers with 47.7% sensitivity, 81.3% specificity, 93.5% positive predictive value and 22.0% negative predictive value. Among the 88 patients surviving on day 3, defective TNF-α production less than 250 pg/ml was found in 42; 8 (19.0%) of them died, as opposed to 2 (4.3%) of 46 patients in whom TNF-α was above 250 pg/ml (*p* = 0.043). The OR for death with defective production of TNF-α by PBMCs after stimulation with LPS was 5.18 (95% CI 1.03–25.97, *p* = 0.046); this was also shown after survival analysis (Fig. 4c).

These findings drive the concept that defective production of TNF-α by PBMCs on day 3 may be a marker of sepsis-induced immunosuppression and generate a key question: Can clinical signs of a patient on admission guide the prediction of the level of TNF-α production by PBMCs on day 3? To answer this question, univariate analysis was performed using demographic and clinical characteristics of patients with TNF-α production greater than 250 pg/ml and TNF-α production less than or equal to 250 pg/ml from circulating PBMCs after LPS stimulation on day 3 (Table 2). Differences with *p* values less than 0.100 were found for APACHE II score, sex, acute coagulopathy and CV failure. Sex, acute coagulopathy and CV failure were entered into the equation of the step-wise logistic regression analysis as independent variables. APACHE II was not entered into the equation, because blood pressure is one of the APACHE II components. This analysis revealed that CV failure was the only risk factor associated with defective production of TNF-α on day 3 (Table 3).

**Table 2:** Univariate analysis of characteristics of 88 patients on admission with sepsis in relation to the effected tumour necrosis factor-α production on day 3 by peripheral blood mononuclear cells after stimulation with bacterial lipopolysaccharide

	TNF-α production ≤250 pg/ml (*n* = 42)	TNF-α production >250 pg/ml (*n* = 46)	*p* Value
Male sex, *n* (%)	16 (38.1)	26 (56.5)	0.093
Age, years mean ± SD	69.7 ± 12.2	69.6 ± 19.6	0.755
APACHE II score, mean ± SD	15.36 ± 8.13	11.63 ± 7.20	0.025
PaO_2_/FiO_2_, mmHg, mean ± SD	322.6 ± 150.5	302.5 ± 99.9	0.570
White blood cells, count/mm^3^, mean ± SD	13,735.0 ± 7725.3	15,007.2 ± 7102.4	0.418
Lymphocytes, count/mm^3^, mean ± SD)	1170.0 ± 697.2	1218.1 ± 644.2	0.805
C-reactive protein, mg/L, mean ± SD	152.2 ± 104.3	110.0 ± 90.6	0.135
Type of infection, *n* (%)			
Acute pyelonephritis	17 (40.5)	23 (50.0)	0.399
Acute intra-abdominal infection	16 (38.1)	13 (28.3)	0.370
Gram-negative bacteraemia	9 (21.4)	10 (21.7)	0.901
Type of failing organ, *n* (%)			
Acute respiratory distress syndrome	5 (10.9)	9 (21.4)	0.245
Acute coagulopathy	8 (17.4)	2 (4.8)	0.093
Acute kidney injury	5 (10.9)	4 (9.5)	1.000
Cardiovascular failure	1 (2.2)	9 (21.4)	0.006
At least one chronic disorder, *n* (%)	28 (66.7)	23 (50.0)	0.134
At least one predisposing condition, *n* (%)	15 (35.7)	24 (52.2)	0.138

*Abbreviations: APACHE* Acute Physiology and Chronic Health Evaluation, *PaO*_*2*_ */FiO*_*2*_ Ratio of partial pressure arterial oxygen and fraction of inspired oxygen, *TNF-*α Tumour necrosis factor-α

**Table 3:** Step-wise logistic regression analysis of patients’ characteristics on day 1 to predict sepsis-induced immunosuppression on day 3 as expressed by tumour necrosis factor-α production less than 250 pg/ml after stimulation of peripheral blood mononuclear cells with lipopolysaccharide

	OR	95% CI	*p* Value
Male sex	^a^		0.099
Acute coagulopathy	^a^		0.057
Cardiovascular failure	12.27	1.48–101.66	0.020

^a^These two variables were excluded from the first step of the analysis as being non-significant in the equation

## Discussion

The present study shows that defective cytokine production from circulating PBMCs following a severe gram-negative infection is a major trait of sepsis-induced immunosuppression. Defective TNF-α production after stimulation with LPS is the most susceptible trait of this process and remains as late as day 10, whereas defective TNF-α production induced by stimulation with Pam_3_Cys is restored by day 7. Defective TNF-α production after stimulation with LPS is associated with unfavourable outcome. CV failure on the first day is a predictive sign of the persistence of defective TNF-α production on day 3.

Although traditionally conceived of as a regulator of an exaggerated immune response, it is now broadly accepted that immunosuppression may exist right from the onset of sepsis [2]. This is not the first study showing an association of cytokine production by circulating PBMCs over the course of sepsis with final outcome. Several years ago, Munoz et al. [8] showed a profound inhibition of circulating monocytes for the production of TNF-α and IL-1β. This was most pronounced when LPS was used as a stimulus in patients experiencing gram-negative infections. A rebound of this phenomenon was associated with favourable outcome. We enrolled a larger population than that in the study of Munoz et al. [8]; our studied population had gram-negative bacteria infections, whereas theirs had infections of mixed causality; and we elaborated significant clinical variables that can predict from the first day the likelihood of incapacity of PBMCs for the production of TNF-α. The correlation of diminished TNF-α production and shock was demonstrated in 2001 by de Werra et al. [9]. They compared TNF-α production and CD14 expression on monocytes among patients with severe sepsis, septic shock or cardiogenic shock as well as in healthy volunteers. Their results demonstrated a defective response of monocytes and lower TNF-α levels in both patients with sepsis and patients with cardiogenic shock compared with healthy control subjects.

In a recent publication, Santos et al. [10] stimulated whole blood from 34 patients with sepsis with LPS, *Pseudomonas aeruginosa* and *Staphylococcus aureus*. By flow cytometric analysis, they showed that the intracellular levels of IL-6 and TNF-α in patients with sepsis were significantly lower than those of healthy control subjects. Analysis of a subset of 15 patients after 7 days showed an increase of intracellular cytokine levels. However, they studied fewer patients than we did; they did not report differences between survivors and non-survivors on day 7; and their population comprised patients with infections of mixed aetiology, including the respiratory tract. The same group of researchers [11] focused on serial changes of gene expression of PBMCs in a small number of patients with sepsis developing in the field of community-acquired pneumonia. The level of expression of genes encoding for cytokines involved in the inflammatory response, such as *TNF*, *IL-6* and *IL-8*, was decreased on day 7 among non-survivors compared with the first 48 h from organ dysfunction. This finding was compatible with our observation of persistence of sepsis-induced immunosuppression among non-survivors.

It should be emphasised that the enrolled studied population had sepsis originating from gram-negative bacteria (Table 1). Although gram-negative bacteria carry a wide variety of pathogen-associated molecular patterns (PAMPs) that can interact with Toll-like receptors (TLRs) embedded on circulating monocytes and tissue macrophages, their LPS is the best studied PAMP. Exposure to LPS leads to endotoxin tolerance whereby a second exposure to LPS elicits blurred cytokine production [12–14]. Circulating PBMCs of both survivors and non-survivors have defective cytokine production on day 1 after exposure to LPS, representing signs of LPS tolerance. LPS tolerance down-regulates the stimulatory efficacy of other TLRs [15], and this can partly explain why circulating PBMCs of patients did not respond to stimulation with Pam_3_Cys, which is a purified ligand for TLR1 and TLR2. Owing to the interaction of TLR4 becoming tolerant to LPS with the other TLRs making them tolerant to their ligands, it becomes evident that survivors of our study escaped from tolerance to both TLR2 and TLR4 as early as 48 h after the first blood sampling.

Two main limitations of our study should be underscored. The first limitation is related to the inability to isolate the exact pathogen in all enrolled pathogens, raising concerns regarding whether the results may be generalisable to all patients with severe gram-negative infections. However, the infections to be studied were selected because they are caused mainly by gram-negative bacteria [6], whereas the offending pathogen, wherever isolated, was one gram-negative species. The second limitation is the use of PBMCs that are a mixed population of lymphocytes and monocytes; the lymphocyte/monocyte ratio may change over time, at least between baseline and day 7 [16], and this may hamper precise interpretation of findings.

Our results encourage former attempts with the use of immunostimulation to improve sepsis outcomes. Recombinant human IFN-γ was the most common approach, originating as an idea in the early 1980s [3]. Leentjens et al. [17] conducted a double-blind, placebo-controlled, randomised study with healthy volunteers becoming tolerant to LPS after the intravenous administration of LPS; subcutaneous treatment with IFN-γ reversed LPS tolerance, as shown by increased circulating TNF-α. Other proposed approaches targeting reversal of immunoparalysis include GM-CSF administration [18], recombinant human IL-7 [19, 20] and the anti-PD-1 antibody [21]. Proving the clinical efficacy of these agents requires the conduct of randomised clinical trials where patients most likely to jump into immunosuppression that does not resolve early should be enrolled. The present study provides evidence that patients with CV failure on SIRS onset are most likely to have persistence of sepsis-induced immunosuppression on day 3. These patients may be likely for inclusion in studies with agents targeting reversal of immunosuppression.

## Conclusions

The present study emphasises the association between cytokine stimulation of circulating PBMCs and final outcome. Sustained decreased cytokine production is linked with great risk for unfavourable outcome, and it is a characteristic of sepsis-induced immunosuppression. Defective production of TNF-α lasts as long as day 10, and it is associated with greatest risk for unfavourable outcome. CV failure on admission is a major indicator of the likelihood for defective TNF-α responses over the time course of sepsis. These findings can help in the design of future trials on immunostimulation for the management of severe sepsis.

## Additional files

Additional file 1: Table S1. Rationale for the selection of measured cytokines in supernatants of PBMCs. (DOCX 13 kb)
Additional file 2: Table S2.
*p* Values of comparisons between TNF-α production by PBMCs of healthy control subjects and patients with sepsis in relation to their 28-day outcomes. *p* Values are corrected according to the method of Bonferroni. (DOCX 14 kb)

## Abbreviations

APACHE: Acute Physiology and Chronic Health Evaluation
CV: Cardiovascular
GM-CSF: Granulocyte-macrophage colony-stimulating factor
IFN-γ: Interferon-γ
IL: Interleukin
LPS: Lipopolysaccharide
PAMP: Pathogen-associated molecular pattern
PaO_2_/FiO_2_: Ratio of partial pressure arterial oxygen and fraction of inspired oxygen
PBMC: Peripheral blood mononuclear cell
SIRS: Systemic inflammatory response syndrome
SOFA: Sequential Organ Failure Assessment
TLR: Toll-like receptor
TNF-α: Tumour necrosis factor-α

## References

[1] Stevenson EK, Rubenstein AR, Radin GT, Wiener RS, Walkey AJ (2014). Two decades of mortality trends among patients with severe sepsis: a comparative meta-analysis. Crit Care Med.

[2] Hotchkiss RS, Monneret G, Payen D (2013). Sepsis-induced immunosuppression: from cellular dysfunctions to immunotherapy. Nat Rev Immunol.

[3] Giamarellos-Bourboulis EJ (2010). What is the pathophysiology of the septic host upon admission?. Int J Antimicrob Agents.

[4] Reinhart K, Bauer M, Riedemann NC, Hartog CS (2012). New approaches to sepsis: molecular diagnostics and biomarkers. Clin Microbiol Rev.

[5] Boomer JS, To K, Chang KC, Takasu O, Osborne DF, Walton AH (2011). Immunosuppression in patients who die of sepsis and multiple organ failure. JAMA.

[6] Giamarellos-Bourboulis EJ, Mylona V, Antonopoulou A, Tsangaris I, Koutelidakis I, Marioli A (2014). Effect of clarithromycin in patients with suspected Gram-negative sepsis: results of a randomized controlled trial. J Antimicrob Chemother.

[7] Singer M, Deutschman CS, Seymour CW, Shankar-Hari M, Annane D, Bauer M (2016). The Third International Consensus Definitions for Sepsis and Septic Shock (Sepsis-3). JAMA.

[8] Munoz C, Carlet J, Fitting C, Misset B, Blériot JP, Cavaillon JM (1991). Dysregulation of in vitro cytokine production by monocytes during sepsis. J Clin Invest.

[9] de Werra I, Zanetti G, Jaccard C, Chioléro R, Schaller MD, Yersin B (2001). CD14 expression on monocytes and TNFα production in patients with septic shock, cardiogenic shock or bacterial pneumonia. Swiss Med Wkly.

[10] Santos SS, Carmo AM, Brunialti MKC, Machado FR, Azevedo LC, Assunção M (2016). Modulation of monocytes in septic patients: preserved phagocytic activity, increased ROS and NO generation, and decreased production of inflammatory cytokines. Intensive Care Med Exp.

[11] Severino P, Silva E, Baggio-Zappia GL, Brunialti MKC, Nucci LA, Rigato O (2014). Patterns of gene expression in peripheral blood mononuclear cells and outcomes from patients with sepsis secondary to community acquired pneumonia. PLoS One.

[12] Martin C, Boisson C, Haccoun M, Thomachot L, Mege JL (1997). Patterns of cytokine evolution (TNF-α and IL-6) after septic shock, hemorrhagic shock, and severe trauma. Crit Care Med.

[13] Morris MC, Gilliam EA, Li L (2015). Innate immune programming by endotoxin and its pathological consequences. Trends Immunol.

[14] Kopanakis K, Tzepi IM, Pistiki A, Carrer DP, Netea MG, Georgitsi M (2013). Pre-treatment with low dose endotoxin prolongs survival from experimental lethal endotoxic shock: benefit for lethal peritonitis by *Escherichia coli*. Cytokine.

[15] de Vos AF, Pater JM, Van den Pangaart PS, de Kruif MD, Van ’t Veer C, Van der Poll T (2009). In vivo lipopolysaccharide exposure of human blood leukocytes induces cross-tolerance to multiple TLR ligands. J Immunol.

[16] Payen D, Laukaszewicz AC, Belikova I, Faiver V, Gelin C, Russwurm S (2008). Gene profiling in human leukocytes during recovery from septic shock. Intensive Care Med.

[17] Leentjens J, Kox M, Koch RM, Preijers F, Joosten LA, van der Hoeven JG (2012). Reversal of immunoparalysis in humans in vivo. Am J Respir Crit Care Med.

[18] Meisel C, Schefold JC, Pschowski R, Baumann T, Hetzger K, Gregor J (2009). GM-CSF to reverse sepsis-associated immunosuppression: a double-blind, randomized, placebo-controlled multicenter trial. Am J Respir Crit Care Med.

[19] Mackall CL, Fry TJ, Gress RE (2009). Harnessing the biology of IL-7 for therapeutic application. Nat Rev Immunol.

[20] Unsinger J, McGlynn M, Kasten KR, Hoekzema AS, Watanabe E, Muenzer JT (2010). IL-7 promotes T cell viability, trafficking, and functionality and improves survival in sepsis. J Immunol.

[21] Brahmamdam P, Inoue S, Unsinger J, Chang KC, McDunn JE, Hotchkiss RS (2010). Delayed administration of anti-PD-1 antibody reverses immune dysfunction and improves survival during sepsis. J Leukoc Biol.

