# Supplementary material for: Decreased cytokine production by mononuclear cells after severe gram-negative infections: early clinical signs and association with final outcome
Source: Crit Care. 2017 Mar 9;21:48. doi: 10.1186/s13054-017-1625-1 (PMC5343541; doi:10.1186/s13054-017-1625-1)
Supplement: Additional file 1: Table S1. — Rationale for the selection of measured cytokines in supernatants of PBMCs. (DOCX 13 kb) [file 13054_2017_1625_MOESM1_ESM.docx]

**Additional file 1: Table S1 Rationale for the selection of measured cytokines in supernatants of PBMCs**

| **Cytokine** | **Rationale** |
| --- | --- |
| IL-4 48-hour incubation | Expression of T-helper 2 function |
| IL-6 24-hour incubation | Expression of monocyte activation |
| IL-8 24-hour incubation | Expression of monocyte activation |
| IL-10 48-hour incubation | Expression of T-helper 2 function |
| IL-12 48-hour incubation | Expression of antigen-presentation |
| IFNγ 48-hour incubation | Expression of T-helper 1 function |
| GM-CSF 48-hour incubation | Expression of monocyte activation |
| TNFα 24-hour incubation | Expression of monocyte activation |
