# Supplementary material for: Decreased cytokine production by mononuclear cells after severe gram-negative infections: early clinical signs and association with final outcome
Source: Crit Care. 2017 Mar 9;21:48. doi: 10.1186/s13054-017-1625-1 (PMC5343541; doi:10.1186/s13054-017-1625-1)
Supplement: Additional file 2: Table S2. — p Values of comparisons between TNF-α production by PBMCs of healthy control subjects and patients with sepsis in relation to their 28-day outcomes. p Values are corrected according to the method of Bonferroni. (DOCX 14 kb) [file 13054_2017_1625_MOESM2_ESM.docx]

**Additional file 2: Table S2 P-values of comparisons between TNFα production by PBMCs of healthy controls and patients with sepsis in relation to their 28-day outcome; p-values are corrected according to Bonferroni**

| **Day** | **Comparison** | **p-value** | **Comparison** | **p-value** |
| --- | --- | --- | --- | --- |
| Day 1 | Healthy controls vs survivors  LPS stimulation | 1.4 x 10^-4^ | Healthy controls vs survivors Pam3Cys stimulation | 0.007 |
|  | Healthy controls vs non-survivors LPS stimulation | 0.007 | Healthy controls vs non-survivors Pam3Cys stimulation | 0.014 |
| Day 3 | Healthy controls vs survivors  LPS stimulation | 2.5 x 10^-3^ | Healthy controls vs survivors Pam3Cys stimulation | 0.273 |
|  | Healthy controls vs non-survivors LPS stimulation | 0.001 | Healthy controls vs non-survivors Pam3Cys stimulation | 0.623 |
| Day 7 | Healthy controls vs survivors  LPS stimulation | 2.3 x 10^-3^ | Healthy controls vs survivors Pam3Cys stimulation | 0.084 |
|  | Healthy controls vs non-survivors LPS stimulation | 0.007 | Healthy controls vs non-survivors Pam3Cys stimulation | 0.787 |
| Day 10 | Healthy controls vs survivors  LPS stimulation | 6.9 x 10^-3^ | Healthy controls vs survivors Pam3Cys stimulation | 0.028 |
|  | Healthy controls vs non-survivors LPS stimulation | 0.089 | Healthy controls vs non-survivors Pam3Cys stimulation | 0.882 |
